# Which Coronary Lesions Are More Prone to Cause Acute Myocardial Infarction?

**DOI:** 10.5935/abc.20170003

**Published:** 2017-02

**Authors:** Taner Sen, Mehmet Ali Astarcioglu, Osman Beton, Lale Dinc Asarcikli, Celal Kilit

**Affiliations:** 1 Dumlupinar University Kutahya Evliya Celebi Education and Research Hospital, Kutahya - Turkey; 2 Sivas Cumhuriyet University, Sivas - Turkey; 3 Diskapi Education and Research Hospital, Ankara - Turkey

**Keywords:** Plaque, Atherosclerotic, Rupture, Myocardial Infarction, Coronary Restenosis, Collateral Circulation

## Abstract

**Background:**

According to common belief, most myocardial infarctions (MIs) are due to the rupture of nonsevere, vulnerable plaques with < 70% obstruction. Data from recent trials challenge this belief, suggesting that the risk of coronary occlusion is, in fact, much higher after severe stenosis. The aim of this study was to investigate whether or not acute ST-elevation MIs result from high-grade stenoses by evaluating the presence of coronary collateral circulation (CCC).

**Methods:**

We retrospectively included 207 consecutive patients who had undergone primary percutaneous coronary intervention for acute ST-elevation MI. Collateral blood flow distal to the culprit lesion was assessed by two investigators using the Rentrop scoring system.

**Results:**

Out of the 207 patients included in the study, 153 (73.9%) had coronary collateral vessels (Rentrop 1-3). The Rentrop scores were 0, 1, 2, and 3 in 54 (26.1%), 50 (24.2%), 51 (24.6%), and 52 (25.1%) patients, respectively. Triglycerides, mean platelet volume (MPV), white cell (WBC) count, and neutrophil count were significantly lower in the group with good collateral vessels (p = 0.013, p = 0.002, p = 0.003, and p = 0.021, respectively).

**Conclusion:**

More than 70% of the patients with acute MI had CCC with Rentrop scores of 1-3 during primary coronary angiography. This shows that most cases of acute MI in our study originated from underlying high-grade stenoses, challenging the common believe. Higher serum triglycerides levels, greater MPV, and increased WBC and neutrophil counts were independently associated with impaired development of collateral vessels.

## Introduction

An ST-segment myocardial infarction (STEMI) is the result of an abrupt rupture of a coronary atherosclerotic plaque and subsequent thrombosis. Most myocardial infarctions (MIs) are thought to follow the rupture of vulnerable plaques deemed nonsevere and with less than 70% obstruction.^1-3^ This belief has been mostly founded on old studies and, as a result, has been debated in recent years. In these older trials, the time between the angiography and the index case was long; this may be problematic since noncritical lesions may progress to a more severe stenosis with time. Reflecting this issue, Alderman et al. published in 1993 a 5-year, prospective, follow-up study (the CASS trial) in which they suggested that the risk of coronary occlusion was much higher in severe compared with nonsevere stenoses.^4^

Collateral vessels develop distally from the ischemic area to compensate for the decreased blood supply distal to the lesions. These collateral vessels may preserve the myocardial function in the case of severe stenosis. In this study, we used the presence of coronary collateral circulation (CCC) as a marker of severe stenosis. Our hypothesis was that the finding of coronary collaterals distal to the culprit lesion would mean that the lesion responsible for the acute occlusion was already severe prior to the episode of acute MI. The aim of this study was to investigate in patients with an episode of acute STEMI whether this episode originated or not from high-grade stenoses.

## Methods

We included retrospectively 207 patients who had undergone primary percutaneous coronary intervention (PCI) due to an acute STEMI at the Dumlupinar University Kutahya Evliya Celebi Education and Research Hospital during a 6 month-period between January 2012 and June 2012. The patients were selected from our catheter laboratory database. At least two physicians double-checked the database to guarantee the accuracy of the data.

The definition of STEMI comprised an ST-segment elevation greater than 1 mm in two or more contiguous precordial leads, or two or more adjacent limb leads, or new or presumed new left bundle-branch block with angina. The culprit lesion was defined as the lesion that received the intervention. Collateral blood flow distal to the culprit lesion was measured by two investigators using the Rentrop grading system:^5^

Rentrop 0 - No visible filling of collateral vessels;
Rentrop 1 - Filling of collateral vessels without any epicardial filling of the artery to be dilated;
Rentrop 2 - Partial epicardial filling by collateral vessels of the artery to be dilated;
Rentrop 3 - Complete epicardial filling by collateral vessels of the artery to be dilated.

Only patients who had undergone primary PCI for acute STEMI were included in the study. Patients with acute coronary syndromes without ST elevation and those who did not undergo primary PCI were excluded.

The number and percentages of the patients who had CCC according to the Rentrop scoring system were calculated. We then divided the patients into two groups according to the rating of the collateral vessels and the Rentrop scores: patients with collateral vessels deemed "poor" (Rentrop 0-1) were included in group 1 and those with collateral vessels deemed "good" (Rentrop 1-3) were included in group 2.

### Statistical analysis

Continuous variables are expressed as mean ± standard deviation (SD) and categorical variables are presented as numbers and percentages. The normality of the data was tested with the Kolmogorov-Smirnov test. Numerical predictors were estimated with the Mann-Whitney U test, whereas categorical predictors were estimated with Pearson's chi-square test. Differences were considered statistically significant when p < 0.05. The variables with a p value below 0.1 were included in a multiple logistic regression analysis.

## Results

Out of the 207 patients included in the study, 138 were males (67%) and 69 were females (33%). The mean age of the patients was 63 ± 11 years. In total, 153 patients (73.9%) presented CCC (Rentrop 1-3). The Rentrop scores were 0, 1, 2, and 3 in 54 (26.1%), 50 (24.2%), 51 (24.6%), and 52 (25.1%) patients, respectively (Table 1). The left anterior descending artery was the most common culprit artery (48.3%), followed by the right coronary artery (30.9%), and the circumflex artery (20.8%). The most common acute STEMI type was inferior MI (52%).

**Table 1:** Distribution of the patients according to Rentrop scores

Rentrop score	Number of patients	%
0	54	26,1
1	50	24,2
2	51	24,6
3	52	25,1
1-3	153	73,9

When we grouped the patients according to the adequacy of collateral vessel development as "poor" (Rentrop 0-1) and "good" (Rentrop 2-3), we found no significant differences between these groups in terms of baseline demographic and clinical characteristics. Triglycerides, mean platelet volume (MPV), white cell (WBC) count, and neutrophil count were significantly lower in the group with "good" collateral vessel development (p = 0.013, p = 0.002, p = 0.003 and p = 0.021, respectively).

Multiple logistic regression analysis showed that triglycerides levels (odds ratio [OR] 1.005, 95% confidence interval [95%CI] 1.001-1.008), MPV (OR 1.271, 95%CI 1.084-1.490), WBC count (OR 1.142, 95%CI 1.020-1.278), and neutrophil count (OR 1.159, 95%CI 1.040-1.292) were independent predictors of CCC (Table 2).

**Table 2:** Univariate and multivariate predictors of inadequate coronary collateral circulation (CCC, Rentrop 0 and 1)

Variable	Univariate analysis				Multivariate analysis		
	**OR **	**p **	**95%CI **		**OR **	**p **	**95%CI **
Triglycerides (TG)	(TG)	0.006	1.001-1.008		1.004	0.018	1.001-1.008
Mean platelet volume (MPV)	1.271	0.003	1.084-1.490		1.215	0.021	1.030-1.434
Neutrophil count	1.159	0.007	1.040-1.292				
White cell (WBC) count	1.142	0.022	1.020-1.278		1.142	0.020	1.021-1.278

TG, MPV, and neutrophil and WBC count were analyzed with forward stepwise multiple logistic regression. CI: confidence interval; OR: odds ratio.

## Discussion

Our study showed that the majority of the acute STEMIs originated from severe stenotic segments of coronary arteries. A total of 73.9% of our patients had coronary collateral vessels, indicating that the majority of the acute MIs originated from previous severe stenotic lesions. This finding challenges the historical belief that acute MI occurs as a result of abrupt rupture of nonsignificant (< 50% obstruction) coronary lesions.

This is a field with many controversies. Older studies supported the idea that coronary occlusion and acute STEMI due to sudden plaque rupture occur from nonsignificant coronary stenotic lesions.^6,7^ Little et al.^1^ conducted one such study in which they monitored 29 patients after coronary angiography until they presented MI. The mean follow-up time was 706 days. As a result, the initial stenosis was below 70% in 97% of the patients. They concluded that the majority of the cases of MI arose from nonsignificant coronary stenosis.^1^ The major limitation of their study was that the time from the initial angiography to the acute MI was so long that nonsignificant coronary lesions could have progressed to high-grade stenosis during follow-up. In another study by Hackett et al.,^8^ the authors found that the mean residual stenosis was below 70% in patients with acute MI after successful thrombolytic therapy. In 1993, Alderman et al. reported results of a prospective study showing that severe lesions were more likely to progress to total occlusion than mild ones after a follow-up period of 5 years.^4^

Results of more recent studies dispute these findings. Frobert et al.^9^ conducted a study in 156 patients with MI who had spontaneous reflow or reflow after uncomplicated wiring at the first angioplasty. Using quantitative coronary analysis (QCA) programs to measure the severity of the culprit lesion, they found that the severity of the underlying lesion was > 50% in 151 (96%) patients and > 70% in 103 (66%) of them.^9^ However, the main disadvantage of this method is that it excludes the presence of thrombus, since the presence of thrombi makes the lesion appear more severe than they really are. Manoharan et al.^10^ performed thrombus aspiration after wiring the culprit lesions in patients with STEMI undergoing primary coronary angioplasty. They then measured the severity of the underlying coronary stenosis with QCA and found that only 11% of the culprit stenoses were below 50%.^10^

In our study, we used the presence of CCC and the Rentrop scoring system to assess the severity of the underlying lesions, instead of using thrombolytic application, thrombus aspiration, and recanalization (spontaneous or wiring), as done in other previous studies.

In a study similar to ours, Khoo et al.^11^ investigated the development of collateral vessels using the Rentrop grading system in 159 patients with acute MI. Of all patients, 95 (60%) had collateral vessels.^11^ Their study supports our findings and was the first trial using CCC as a surrogate marker for underlying lesion severity. Our study is the second trial using this method but our sample size is larger than that in the study by Khoo et al. ^11^

Collateral vessels are vascular connections from one coronary vessel to other high-grade, stenotic vessels.^12^ This is an adaptation to ischemia. Although the exact mechanism for this occurrence is unknown, it has been suggested to be through the release of some growth factors in response to ischemia.^13^ Collateral vessels have some beneficial effects, including reduced infarct size, preservation of ejection function, and reduction of postinfarction complications like rupture and aneurysm.^14-16^ While coronary collaterals may supply enough blood flow during rest, they may not supply sufficient flow during exercise.^17^

The degree of collateral development varies among patients. It is not clear why some patients have a Rentrop score of 3 for collateral vessels, while others have a Rentrop 1 score. Several factors and markers have been identified as contributors to the development of coronary collateral vessels. The severity of the underlying coronary stenosis, proximal location of the lesion, symptom duration, and slow heart rates are described as clinical factors that influence the development of collaterals.^18-20^ Granulocyte-monocyte-colony stimulating factor (GM-CSF) and granulocyte-colony stimulating factor (G-CSF), physical exercise, and external counterpulsation have also been found to positively affect the development of collaterals, whereas aging, obesity, and levels of uric acid and C-reactive protein have been found to have negative effects.^21-28^

We found in our study that higher levels of serum triglycerides, greater MPV, and increased WBC and neutrophil counts were independently associated with impairment of collateral vessel development. Akın et al.^29^ reported that the level of serum triglycerides and ratio of neutrophil / lymphocyte (N/L) were independently associated with poor CCC development after multivariate regression analysis. MPV and WBC count were not different between the groups with poor and good CCC in their study. In our study, we did not find any significant association between N/L ratio and CCC, expect for the neutrophil count.

The association between MPV and CCC is unclear. Ege et al.^30^ reported that MPV levels were significantly higher in patients with poor CCC and coronary artery disease (CAD). In contrast, Duran et al. reported that elevated MPV levels were independent predictors of a good CCC development in patients with acute coronary syndrome.^31^ While Kadı et al. found that levels of high-density cholesterol (HDL-C) were associated with good CCC development,^32^ we found that serum triglycerides level was positively associated with CCC development.

The presence of coronary collateral vessels may imply that the underlying stenosis is severe. In our study, we regarded patients with Rentrop 1-3 coronary collateral vessel development as having underlying high-grade ischemia causing stenosis. Our use of collateral vessel development as a surrogate marker of ischemia may reflect more reliably the physiological reality than methods to measure the anatomical calculation of lesion severity used in previous studies.

### Study limitations

Collateral vessels of small caliber may not have been visible during coronary angiography. With that, we may have underestimated the presence of coronary collateral vessels.

The Rentrop scoring system is a subjective method to evaluate collateral vessel development. Coronary flow index is a better method for this evaluation, as it is a more objective and sensitive technique to determine the development of CCC. However, while it may evaluate CCC more accurately than Rentrop, it is an invasive technique and not easy to incorporate into routine clinical practice.

## Conclusion

Most cases of acute myocardial ischemia originated from underlying high-grade stenoses, contrary to older belief. More than 70% of the patients with acute MI had CCC with Rentrop scores of 1-3 during primary coronary angioplasty. Higher serum triglycerides level, greater MPV, and increased WBC and neutrophil counts were independently associated with impairment of collateral vessel development.

## References

[1] Little WC, Constantinescu M, Applegate RJ, Kutcher MA, Burrows MT, Kahl FR (1988). Can coronary angiography predict the site of a subsequent
myocardial infarction in patients with mild-to-moderate coronary artery
disease?. Circulation.

[2] Giroud D, Li JM, Urban P, Meier B, Rutishauer W (1992). Relation of the site of acute myocardial infarction to the most
severe coronary arterial stenosis at prior angiography. Am J Cardiol.

[3] Ambrose JA, Tannenbaum MA, Alexopoulos D, Hjemdahl-Monsen CE, Leavy J, Weiss M (1988). Angiographic progression of coronary artery disease and the
development of myocardial infarction. J Am Coll Cardiol.

[4] Alderman EL, Corley SD, Fisher LD, Chaitman BR, Faxon DP, Foster ED (1993). Five-year angiographic follow-up of factors associated with
progression of coronary artery disease in the Coronary Artery Surgery Study
(CASS). CASS Participating Investigators and Staff. J Am Coll Cardiol.

[5] Rentrop KP, Cohen M, Blanke H, Phillips RA (1985). Changes in collateral filling immediately after controlled
coronary artery occlusion by an angioplasty balloon in human
subjects. J Am Coll Cardiol.

[6] Shah PK (2003). Mechanisms of plaque vulnerability and rupture. J Am Coll Cardiol.

[7] Falk E, Shah PK, Fuster V (1995). Coronary plaque disruption. Circulation.

[8] Hackett D, Davies G, Maseri A (1988). Pre-existing coronary stenoses in patients with first myocardial
infarction are not necessarily severe. Eur Heart J.

[9] Frobert O, van't Veer M, Aarnoudse W, Simonsen U, Koolen JJ, Pijls NH (2007). Acute myocardial infarction and underlying stenosis
severity. Catheter Cardiovasc Interv.

[10] Manoharan G, Ntalianis A, Muller O, Hamilos M, Sarno G, Melikian N (2009). Severity of coronary arterial stenoses responsible for acute
coronary syndromes. Am J Cardiol.

[11] Khoo V, Shen L, Zhao L, Khoo V, Loo G, Richards AM (2014). Determination of the severity of underlying lesions in acute
myocardial infarction on the basis of collateral vessel
development. Coron Artery Dis.

[12] Tayebjee MH, Lip GY, MacFadyen RJ (2004). Collateralization and the response to obstruction of epicardial
coronary arteries. QJM.

[13] Fujita M, Ikemoto M, Kishishita M, Otani H, Nohara R, Tanaka T (1996). Elevated basic fibroblast growth factor in pericardial fluid of
patients with unstable angina. Circulation.

[14] Habib GB, Heibig J, Forman SA, Brown BG, Roberts R, Terrin ML (1991). Influence of coronary collateral vessels on myocardial infarct
size in humans: results of phase I thrombolysis in myocardial infarction
(TIMI) trial. The TIMI Investigators. Circulation.

[15] Nicolau JC, Pinto MA, Nogueira PR, Lorga AM, Jacob JL, Garzon SA (1997). The role of antegrade and collateral flow in relation to left
ventricular function post-thrombolysis. Int J Cardiol.

[16] Hirai T, Fujita M, Nakajima H, Asanoi H, Yamanishi K, Ohno A (1989). Importance of collateral circulation for prevention of left
ventricular aneurysm formation in acute myocardial
infarction. Circulation.

[17] Bache RJ, Schwartz JS (1983). Myocardial blood flow during exercise after gradual coronary
occlusion in the dog. Am J Physiol.

[18] Piek JJ, van Liebergen RA, Koch KT, Peters RJ, David GK (1997). Clinical, angiographic and hemodynamic predictors of recruitable
collateral flow assessed during balloon angioplasty coronary
occlusion. J Am Coll Cardiol.

[19] Werner GS, Ferrari M, Betge S, Gastmann O, Richartz BM, Figulla HR (2001). Collateral function in chronic total coronary occlusions is
related to regional myocardial function and duration of
occlusion. Circulation.

[20] de Marchi SF, Gloekler S, Meier P, Traupe T, Steck H, Cook S (2011). Determinants of preformed collateral vessels in the human heart
without coronary artery disease. Cardiology.

[21] Seiler C, Pohl T, Wustmann K, Hutter D, Nicolet PA, Windecker S (2001). Promotion of collateral growth by granulocyte-macrophage
colony-stimulating factor in patients with coronary artery disease: a
randomized, double-blind, placebo-controlled study. Circulation.

[22] Meier P, Gloekler S, de Marchi S, Indermuehle A, Rutz T, Traupe T (2009). Myocardial salvage through coronary collateral growth by
granulocyte colony-stimulating factor in chronic coronary artery disease: a
controlled randomized trial. Circulation.

[23] Zbinden R, Zbinden S, Meier P, Hutter D, Billinger M, Wahl A (2007). Coronary collateral flow in response to endurance exercise
training. Eur J Cardiovasc Prev Rehabil.

[24] Gloekler S, Meier P, de Marchi SF, Rutz T, Traupe T, Rimoldi SF (2010). Coronary collateral growth by external counterpulsation: a
randomised controlled trial. Heart.

[25] Epstein SE, Lassance-Soares M, Faber JE, Burnett MS (2012). Effects of aging on the collateral circulation, and therapeutic
implications. Circulation.

[26] Duran M, Ornek E, Murat SN, Turfan M, Vatankulu MA, Ocak A (2012). High levels of serum uric acid impair development of coronary
collaterals in patients with acute coronary syndrome. Angiology.

[27] Kadı H, Ceyhan K, Karayakalı M, Koç F, Celik A, Onalan O (2011). The relationship between coronary collateral circulation and
blood high-sensitivity C-reactive protein levels. Turk Kardiyol Dern Ars.

[28] Yilmaz MB, Biyikoglu SF, Akin Y, Guray U, Kisacik HL, Korkmaz S (2003). Obesity is associated with impaired coronary collateral vessel
development. Int J Obes Relat Metab Disord.

[29] Akın F, Ayça B, Çelik Ö, Şahin C (2015). Predictors of poor coronary collateral development in patients
with stable coronary artery disease: neutrophil-to-lymphocyte ratio and
platelets. Anatol J Cardiol.

[30] Ege MR, Acıkgoz S, Zorlu A, Sıncer I, Guray Y, Guray U (2013). Mean platelet volume: an important predictor of coronary
collateral development. Platelets.

[31] Duran M, Gunebakmaz O, Uysal OK, Ocak A, Yilmaz Y, Arinc H (2013). Relation between mean platelet volume and coronary collateral
vessels in patients with acute coronary syndromes. J Cardiol.

[32] Kadı H, Özyurt H, Ceyhan K, Koç F, Çelik A, Burucu T (2012). The relationship between high-density lipoprotein cholesterol and
coronary collateral circulation in patients with coronary artery
disease. J Investig Med.

